# Acute Proptosis in a Diabetic Patient: Diagnostic and Therapeutic Dilemmas

**DOI:** 10.7759/cureus.15580

**Published:** 2021-06-10

**Authors:** Ibrahim Khairul-Anwar, Evelyn Tai, Adil Hussein, Embong Zunaina

**Affiliations:** 1 Department of Ophthalmology and Visual Science, School of Medical Sciences, Universiti Sains Malaysia, Kubang Kerian, MYS; 2 Department of Ophthalmology, Hospital Universiti Sains Malaysia, Kubang Kerian, MYS; 3 Department of Ophthalmology, Kulliyyah of Medicine, International Islamic University of Malaysia, Kuantan, MYS; 4 Department of Ophthalmology and Visual Science, School of Medical Sciences, Universiti Sains Malaysia, Kota Bharu, MYS

**Keywords:** proptosis, idiopathic orbital inflammatory disease, orbital cellulitis, corticosteroids

## Abstract

An elderly diabetic lady presented with a painful swollen right eye and blurred vision for one week, preceded by right eye redness for six months. Her right eye best-corrected visual acuity was finger counting at 1 m. There was right eye proptosis, limited extraocular muscle movements, corkscrew vessels, chemosis and elevated intraocular pressure, but no bruit. Fever was absent. Computed tomography of the brain and orbit showed thickened extraocular muscles and intraconal fat streakiness, with normal superior ophthalmic vein and concavity of the cavernous sinus. Intravenous antibiotics resulted in limited clinical improvement. The subsequent response to oral prednisolone was dramatic, with the improvement of visual acuity to 20/60 after three doses. In cases of atypical orbital cellulitis where antibiotics fail, one should consider differential diagnoses such as orbital inflammatory disease, vascular anomalies and masqueraders. We discuss the approach to the diagnosis of acute proptosis in a diabetic patient and highlight the role of corticosteroids in idiopathic orbital inflammatory disease.

## Introduction

Orbital cellulitis is an acute infection, which requires timely diagnosis and therapy to avoid significant visual and life-threatening sequelae such as optic neuropathy, encephalomeningitis, cavernous sinus thrombosis, sepsis and intracranial abscess formation. The mainstay of therapy is intravenous antibiotics [1]. However, in cases where these fail to elicit a satisfactory response, there is a need to consider differential diagnoses such as idiopathic orbital inflammatory disease (IOID), masquerade syndrome and vascular anomalies. We highlight the approach to diagnosis and management of an atypical case of orbital cellulitis.

## Case presentation

An elderly lady with underlying diabetes mellitus, hypertension and hyperlipidemia presented with a painful swollen right eye and blurred vision for one week, preceded by right eye redness for six months. She also complained of nausea but denied any vomiting or tinnitus. There were no constitutional symptoms such as fever or loss of weight.

The best-corrected visual acuity was finger counting at 1 m in the right eye and 20/80 pinhole 20/60 in the left eye. Pupils were 5 mm, fixed, in the right eye and 3 mm, reactive, in the left eye. She had a positive reverse relative afferent pupillary defect in the right eye. Hertel exophthalmometer measured 3 mm of relative proptosis in the right eye, with limitation of extraocular muscle movements in all fields of gaze.

**Figure 1:**
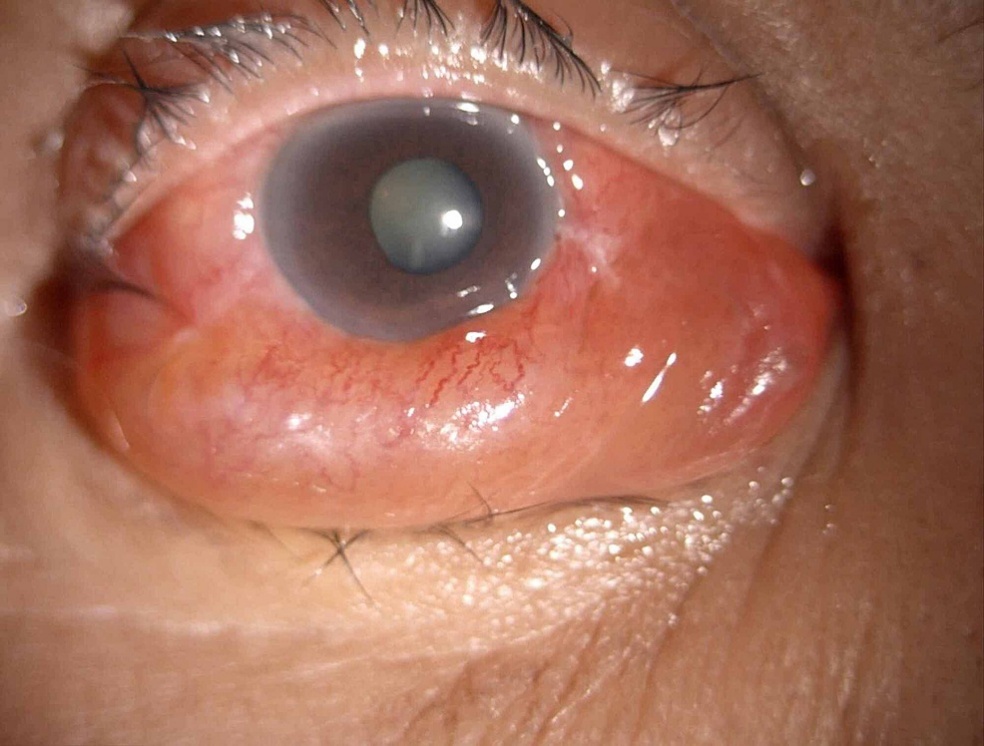
Colour photograph of the right eye demonstrating proptosis, diffuse conjunctival injection, chemosis and corkscrew vessels.

On examination, there was periorbital swelling, mild proptosis, corkscrew vessels, chemosis and diffuse conjunctival injection in the right eye, but no bruit (Figure 1). The remainder of the right eye examination was normal, except for multiple Roth spots along the retinal vascular arcades. The left eye was normal. Intraocular pressure was 58 mmHg in the right eye and 14 mmHg in the left eye. Other cranial nerve examinations were unremarkable. Systemic examination was unremarkable.

**Figure 2:**
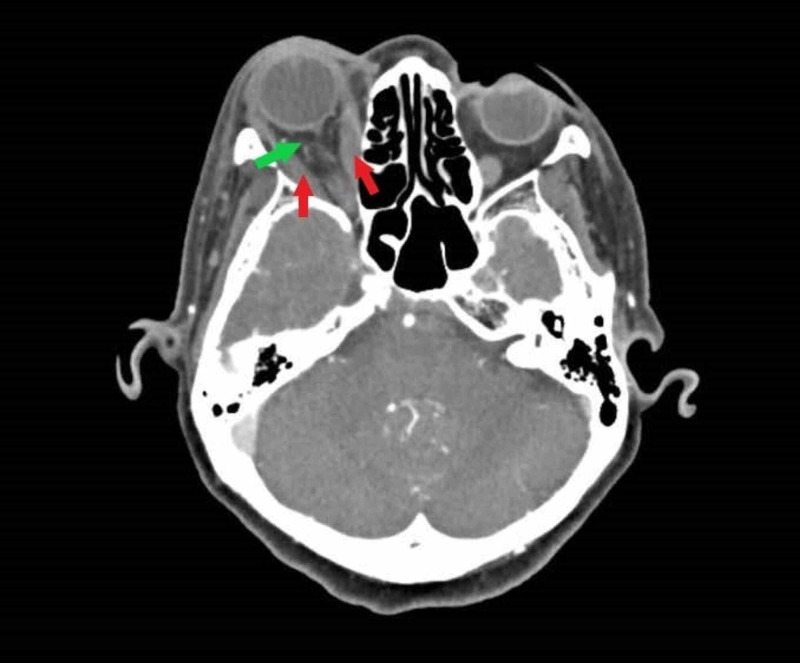
Axial computed tomography of the brain and orbit showing thickened extraocular muscles (red arrow) and streakiness of intraconal fat (green arrow), with normal concavity of the superior ophthalmic vein and cavernous sinus.

Laboratory investigations revealed erythrocyte sedimentation rate of 74 mm/hr with a total white blood cell of 9.75 x 109/L. Computed tomography (CT) of the brain and orbit showed thickened extraocular muscles and streakiness of intraconal fat. There was normal concavity of the superior ophthalmic vein and cavernous sinus, rendering the differential diagnoses of cavernous sinus pathology unlikely (Figure 2). Cultures from blood and conjunctival sac were negative, as was tuberculosis screen. Nasal endoscopy and dental assessment revealed no abnormalities. Her other blood parameters, including thyroid function test and inflammatory markers, were likewise negative.

She was started on intravenous ceftriaxone. Three days later, unasyn (ampicillin with sulbactam) was added in view of limited clinical response. A week after initiating intravenous antibiotics, metronidazole was also prescribed to increase coverage of anaerobic organisms. Despite two weeks of intravenous antibiotics, her right eye proptosis did not resolve, and her vision remained poor. Repeat CT orbit was suggestive of abscess collection at the inferior rectus muscle. Drainage under local anaesthesia was attempted but no pus was present; culture of the swab grew no organisms. As the patient declined biopsy, oral prednisolone 1 mg/kg/day was started empirically. After a mere three doses, her right eye visual acuity improved from finger counting to 20/60, and the relative afferent pupillary defect resolved. She was discharged well after a week in the ward. Oral corticosteroids were subsequently tapered off. Her vision was stable at 20/60 with posterior subcapsular cataract until her last follow up, which was a year after discharge. Her extraocular muscle movement was full and no visual field defect noted.

## Discussion

In a patient with acute proptosis and a congested eye, several differentials come to mind. The differential diagnoses, in this case, included orbital cellulitis (although fever was conspicuously absent), vascular anomalies such as carotid-cavernous fistulas, and IOID. IOID is a term for a group of diseases categorized as non-infective orbital inflammation, which target orbital structures such as lacrimal gland, extraocular muscles and orbital fat. The presentation of the disease depends on the location of the primary orbital inflammation [2]. We highlight a case of orbital inflammatory disease presenting as orbital cellulitis and discuss the differential diagnoses, workup and management of this condition.

As IOID is traditionally a diagnosis of exclusion, comprehensive laboratory testing and radio-imaging are mandatory to rule out other causes, including thyroid ophthalmopathy, although our patient’s clinical features and absence of lid signs render this diagnosis less likely. A CT scan of the orbit is useful not only to see muscle enlargement, which traditionally spares the extraocular muscle tendons in thyroid orbitopathy but also to give clues to the presence of a carotid-cavernous fistula, in which a dilated superior ophthalmic vein will be observed. CT can also detect intraorbital tumours and complications of orbital cellulitis such as subperiosteal or orbital abscesses. CT scan findings suggestive of IOID include diffuse lacrimal gland enlargement in dacryoadenitis, peripheral enhancement of the optic nerve in perineuritis and orbital fatstreakiness [3].

Systemic corticosteroids have been shown to hasten recovery in patients with orbital cellulitis, although the commencement of corticosteroids as the first line of treatment in a patient with a possible infective source is controversial [4]. Many practitioners prefer to “sterilize” the infection with an antibiotic course of appropriate duration before initiating corticosteroids. In orbital cellulitis unresponsive to antibiotics, a repeat CT scan is indicated to look for the presence of any new lesion or focal abscess which may require drainage [4-6].

Empirical treatment with corticosteroids may be a potential cause of diagnostic delay, particularly in cases of malignancy, but in cases of myositic IOID such as ours (in which the CT scan showed enlarged extraocular muscles), a trial of systemic corticosteroids is not unreasonable [1,7]. However, in lesions not confined to the extraocular muscles, the IOID diagnostic criteria consensus recommended a tissue biopsy [8]. This will be useful not only to confirm the diagnosis but also to exclude life-threatening malignancies like lymphoma.

Before initiating systemic immunosuppressive therapy, it is important to screen for contraindications such as tuberculosis, which is endemic in our population. Besides that, a basic metabolic profile is obtained to ensure the patient is systemically fit, as corticosteroids may cause significant metabolic derangements. Our patient’s dramatic response to corticosteroids suggests that in her case, the cause was inflammatory, rather than infective. In the absence of a tissue biopsy, excellent response to corticosteroids is practically pathognomic of IOID, although for improvement to be sustained, a prolonged course with slow taper is required [2].

## Conclusions

Orbital cellulitis is a sight and life-threatening condition which we need to treat and exclude. With poor response to therapy, we need to revise our diagnosis and management plan. Mimics, such as IOID, should be considered after excluding other life- and sight-threatening condition such as mucormycosis, retrobulbar tumour or metastasis. CT scan of the brain and orbit will guide the diagnosis. A dramatic response to corticosteroids is highly suggestive of IOID.

## References

[1] Pushker N, Tejwani LK, Bajaj MS, Khurana S, Velpandian T, Chandra M (2013). Role of oral corticosteroids in orbital cellulitis. Am J Ophthalmol.

[2] Yuen SJ, Rubin PA (2003). Idiopathic orbital inflammation: distribution, clinical features, and treatment outcome. Arch Ophthalmol.

[3] Pakdaman MN, Sepahdari AR, Elkhamary SM (2014). Orbital inflammatory disease: pictorial review and differential diagnosis. World J Radiol.

[4] Lee S, Yen MT (2011). Management of preseptal and orbital cellulitis. Saudi J Ophthalmol.

[5] Eustis HS, Mafee MF, Walton C, Mondonca J (1998). MR imaging and CT of orbital infections and complications in acute rhinosinusitis. Radiol Clin North Am.

[6] Towbin R, Han BK, Kaufman RA, Burke M (1986). Postseptal cellulitis: CT in diagnosis and management. Radiology.

[7] Gavard-Perret A, Lagier J, Delmas J, Delas J, Adenis J-P, Robert P-Y (2015). Rationale for a diagnostic approach in non-Graves’ orbital inflammation--Report of 61 patients. (Article in French). J Fr Ophtalmol.

[8] Mombaerts I, Bilyk JR, Rose GE (2017). Consensus on diagnostic criteria of idiopathic orbital inflammation using a modified Delphi approach. JAMA Ophthalmol.

